# Hijacking the Fusion Complex of Human Parainfluenza Virus as an Antiviral Strategy

**DOI:** 10.1128/mBio.03203-19

**Published:** 2020-02-11

**Authors:** T. C. Marcink, E. Yariv, K. Rybkina, V. Más, F. T. Bovier, A. des Georges, A. L. Greninger, C. A. Alabi, M. Porotto, N. Ben-Tal, A. Moscona

**Affiliations:** a Department of Pediatrics, Columbia University Medical Center, New York, New York, USA; b Center for Host-Pathogen Interaction, Columbia University Medical Center, New York, New York, USA; c Department of Biochemistry and Molecular Biology, George S. Wise Faculty of Life Sciences, Tel Aviv University, Tel Aviv, Israel; d Centro Nacional de Microbiología, Instituto de Salud Carlos III, Madrid, Spain; e CIBER de Enfermedades Respiratorias, Instituto de Salud Carlos III, Madrid, Spain; f Department of Chemistry and Biochemistry, Advanced Science Research Center, City College of New York, New York, New York, USA; g Department of Laboratory Medicine, University of Washington, Seattle, Washington, USA; h Vaccine & Infectious Diseases Division, Fred Hutchinson Cancer Research Center, Seattle, Washington, USA; i Robert Frederick Smith School of Chemical and Biomolecular Engineering, Cornell University, Ithaca, New York, USA; j Department of Experimental Medicine, University of Campania ‘Luigi Vanvitelli,’ Italy; k Department of Microbiology & Immunology, Columbia University Medical Center, New York, New York, USA; l Department of Physiology & Cellular Biophysics, Columbia University Medical Center, New York, New York, USA; Columbia University

**Keywords:** antiviral agents, cryo-electron tomography, viral fusion protein, viral protein structure, viral receptor

## Abstract

The receptor binding protein of parainfluenza virus, hemagglutinin-neuraminidase (HN), is responsible for actively triggering the viral fusion protein (F) to undergo a conformational change leading to insertion into the target cell and fusion of the virus with the target cell membrane. For proper viral entry to occur, this process must occur when HN is engaged with host cell receptors at the cell surface. It is possible to interfere with this process through premature activation of the F protein, distant from the target cell receptor. Conformational changes in the F protein and adoption of the postfusion form of the protein prior to receptor engagement of HN at the host cell membrane inactivate the virus. We previously identified small molecules that interact with HN and induce it to activate F in an untimely fashion, validating a new antiviral strategy. To obtain highly active pretriggering candidate molecules we carried out a virtual modeling screen for molecules that interact with sialic acid binding site II on HN, which we propose to be the site responsible for activating F. To directly assess the mechanism of action of one such highly effective new premature activating compound, PAC-3066, we use cryo-electron tomography on authentic intact viral particles for the first time to examine the effects of PAC-3066 treatment on the conformation of the viral F protein. We present the first direct observation of the conformational rearrangement induced in the viral F protein.

## Abstract (precis)

Paramyxoviruses, including human parainfluenza virus type 3, are internalized into host cells by fusion between viral and target cell membranes. The receptor binding protein, hemagglutinin-neuraminidase (HN), upon binding to its cell receptor, triggers conformational changes in the fusion protein (F). This action of HN activates F to reach its fusion-competent state. Using small molecules that interact with HN, we can induce the premature activation of F and inactivate the virus. To obtain highly active pretriggering compounds, we carried out a virtual modeling screen for molecules that interact with a sialic acid binding site on HN that we propose to be the site involved in activating F. We use cryo-electron tomography of authentic intact viral particles for the first time to directly assess the mechanism of action of this treatment on the conformation of the viral F protein and present the first direct observation of the induced conformational rearrangement in the viral F protein.

## INTRODUCTION

Acute respiratory infection is the leading cause of mortality in children under age 5 years, accounting for ∼20% of childhood deaths worldwide and killing between 2 million and 3 million children each year (1, 2), yet for most of the important viral causes of lower respiratory tract disease, we have no vaccine or drug treatment. The human parainfluenza viruses (HPIVs), along with respiratory syncytial virus (RSV) and human metapneumovirus, cause the majority of cases of childhood croup, bronchiolitis, and pneumonia (3), the three major manifestations of the acute respiratory infections that affect young infants. No vaccines or drugs exist for the HPIVs, HPIV type 1 (HPIV1), HPIV2, HPIV3, or HPIV4 (4), despite the more than 23,000 hospitalizations for HPIV infection yearly in the United States (5). Parainfluenza viruses are a significant cause of lower respiratory tract infection in pediatric and adult patients following hematopoietic stem cell transplantation (HSCT), with HPIV3 being the most important and being associated with high rates of mortality (6–8). In adults with HPIV infection after HSCT, the mortality rate may be 75% (9), and no therapies are effective (8, 10). Recombinant HPIV3 vaccines have passed phase I evaluation, and HPIV1 and HPIV2 vaccine candidates are in pediatric phase I trials (2); however, even this eventual development will not eliminate HPIV disease in the foreseeable future. While the use of corticosteroids has decreased hospitalizations for HPIV1-associated croup (11), HPIV2 and HPIV3 infections remain untreatable, and HPIV3 is responsible for more hospitalizations than HPIV1 and HPIV2 combined (5). Serious HPIV infections urgently need treatment strategies (3, 4, 10).

Parainfluenza virus entry is mediated by fusion of the viral and target host cell membranes at the cell surface. Virus-cell fusion results from the coordinated action of the two envelope glycoproteins that comprise the viral entry complex: the receptor binding protein hemagglutinin-neuraminidase (HN) and the fusion protein (F). These two envelope glycoproteins form a fusion complex and work together to mediate virus attachment and entry into target cells. The HPIV3 HN is a type II transmembrane protein that executes both receptor binding during viral entry and receptor cleavage during viral release from an infected cell. HN also has a dual effect on the F protein: before receptor engagement, HN stabilizes the F protein, but upon receptor engagement, HN activates F (12–14). F is synthesized as a precursor (F_0_) that is cleaved within the cell to yield the prefusion F trimer, with F_1_ and F_2_ remaining covalently linked via a disulfide bond. This trimeric F structure is present on the surface of an infectious viral particle in a metastable prefusion conformation, with its hydrophobic fusion peptide being buried in the interior of the molecule. After HN engages its cell surface sialic acid receptor, it activates F, and the prefusion F undergoes a conformational transition, extending and inserting its hydrophobic fusion peptide N-terminal domain into the target cell (15–20). F proceeds to refold into its energetically stable postfusion structure as the N-terminal and C-terminal complementary heptad repeats meet to form a stable six-helix bundle, and this refolding drives fusion of the viral and cell membranes and release of the viral genetic material into the target cell (4, 16, 17, 19–26).

**FIG 1:**
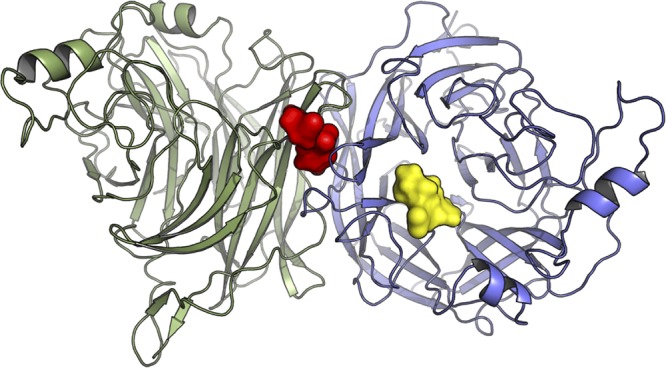
Structural overview of the homodimeric HN protein’s globular head. The two monomers are shown in green and blue. The sialic acid binding sites are highlighted by zanamivir, bound to site I (yellow), and CM9, computationally docked to site II at the dimer interface (red). The figure was generated based on the asymmetrical unit of the protein with PDB accession number 4MZA, using the PyMOL molecular graphics program, version 2.0 (Schrödinger LLC).

The HPIV3 glycoprotein fusion complex is fit for infecting specific human host cells, and the glycoprotein complex of clinical isolate (CI) viruses that are fit for humans differs significantly from that of laboratory-adapted strains of HPIV3 (27–32). HN regulates its several distinct functions via coordination between its cytoplasmic domain, membrane-spanning region, stalk region, and a globular head that contains the primary sialic acid binding site and neuraminidase active site (binding site I), as well as a second sialic acid binding site (site II) that modulates the activation of F (Fig. 1). F itself influences the extent and timing of fusion through its prefusion stability, kinetics of activation, and precursor cleavability. Fusion is moderated through a balance of these functions of HN and F. While viruses bearing the fusion complex of clinical strains grow efficiently in both the human airway and *in vivo* models, they fail to grow on immortalized cells, demonstrating the profound specificity of the HN/F complex for the authentic host (28, 30).

The timing of F activation by HN is key to successful infection. Activation and the subsequent conformational change of the F protein must occur when F is in contact with the target cell membrane. If F assumes its elongated intermediate state or its postfusion state prematurely, the virus is rendered noninfectious (4, 33). The postfusion state of F is irreversible. Molecules that irreversibly inactivate the viral fusion complex, by stimulating HN to trigger F prior to receptor engagement, thus lead to the permanent inactivation of F and the prevention of viral entry (33, 34). We identified several such compounds that induce HN to prematurely activate F, without altering HN’s functions of receptor binding or receptor cleaving (neuraminidase), providing a proof of concept for a new class of antiviral compounds that we refer to as pretriggering or preactivating compounds (33, 34). We also previously described small receptor analog binding inhibitors that target HN’s primary binding site, preventing attachment to cellular receptors (35–38). Here we show that, by combining highly effective pretriggering activity with binding, we obtain synergy and a significant enhancement of inhibitory activity.

To obtain highly active pretriggering candidate molecules, we carried out a virtual modeling screen for molecules that interact with binding site II on HN, which we propose to be the site responsible for activating F. Docking studies revealed a set of molecules with a potential fit at site II on HN’s globular head, of which one (termed premature activating compound 3066, or PAC-3066 from here on) is highly effective. To directly assess the mechanism of action of this compound, we applied cryo-electron tomography to authentic intact viral particles to examine the effects of PAC-3066 treatment on the conformation of the viral F protein, obtaining the first direct observation of the induced conformational rearrangement.

## RESULTS

### Computational evaluation of small molecules that interact with site II of HPIV3 HN.

**FIG 2:**
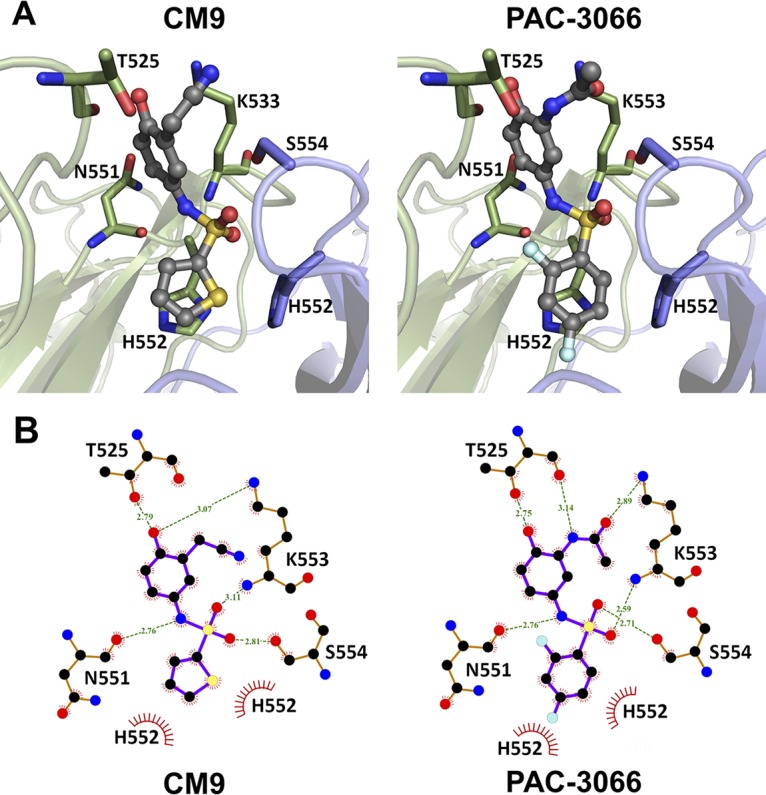
Putative binding modes of HN inhibitors within site II. (A) The (putative) binding poses of the CM9 (left) and PAC-3066 (right) inhibitors. (B) A two-dimensional representation of CM9 (left) and PAC-3066 (right), including their interactions with surrounding amino acids. Green dashed lines represent hydrogen bonds, with the distance between the donor and acceptor heavy atoms being marked. Hydrophobic, stacking interactions with H552 are presented as barbed, red half circles. All atoms are marked using CPK coloring standards, with fluorine being shown in bright green. The two-dimensional figures were prepared using the LigPlot+ program (86).

The structure of HPIV3 HN contains two sialic acid binding sites (Fig. 1). Site I (Fig. 1, yellow) catalyzes the neuraminidase activity of the protein as well as hemagglutinin activity. Site II (Fig. 1, red), which is located in the interface between two HPIV3 HN subunits, promotes activation of the fusion protein (39). While the catalytic site is well resolved in the crystal structure of HPIV3 HN (PDB accession number 4MZA) and has enough space for sialic acid to fit in it, the second site is closed, with only a very small electron density which matches that of an inorganic phosphate filling it. In this compressed conformation, site II is unsuitable for small-molecule docking. An inhibitor of HPIV3 HN, referred to as CSC11, was identified in a previous virtual screen (34). That CSC11 induced fusion protein activation without disrupting either neuraminidase or hemagglutinin activity suggests that it binds to site II. To examine this hypothesis, we initially docked CSC11 using an induced-fit docking protocol (40), which allows for restrained amino acid side chain movement. The induced-fit docking was followed by Glide docking (41) of four additional analogues (including CM9) using the standard precision (SP) energy function. Binding energies for the ligands were calculated using the molecular mechanics energies combined with the generalized born and surface area continuum solvation (MM-GBSA) approach, as described above. The calculated binding energy correlated reasonably well with the observed 50% inhibitory concentration (IC_50_) values (*R*^2^ = 0.76), suggesting that the modeled binding mode and, in particular, the structure of the protein after the induced fit were close to reality. In comparison, when the molecules were docked into site I using the same method, the resulting binding energies correlated very poorly with the IC_50_ values (*R*^2^ = 0.10). As seen by the docking pose of molecule CM9 (Fig. 2, left), the inhibitors fit snugly into site II, with the thiophene ring of CM9 being sandwiched between the two H552 residues (one from each monomer). This histidine residue, along with Q559, is known to constitute a hot spot for the dimerization of HN (27). The sulfonamide group is located in the cavity in which the inorganic phosphate solvent molecule was originally located. The sulfonamide receives hydrogen bonds from S554 and the backbone of K553, while the amide of the group donates a hydrogen bond to the backbone of N551. There was still some room for improvement of existing interactions, as the nitrile group is not optimally aligned to interact with K553. Thus, we used the structure after the induced fit to screen the lead-like subset of the ZINC database (42) against site II, and based on the resulting docking poses and observed interactions, 26 molecules, including PAC-3066 (Fig. 2, right), were selected for experimental assessment.

### Screen of molecules for virucidal activity: a marked enhancement of efficacy.

We initially screened the 26 compounds for a direct and temperature-dependent virucidal effect against clinically relevant strains of virus prior to virus-target cell interaction. For 19 of the 26 compounds, incubation with 5 mM compound at 37°C, a temperature at which F can activate, but not at 4°C, inactivated virus. Of those 26 compounds, 17 were available in an adequate quantity for experimentation and were analyzed further (Table S1 in the supplemental material shows the IC_50_ and IC_90_ values for the entire panel of compounds, as well as toxicity information). We then selected from this group those without cellular toxicity, i.e., those with which cultured cells were viable after exposure to 1 mM compound and those with the most antiviral efficacy both after preincubation of virus and when added during infection. This group of compounds did not damage viral particle integrity (for those that were examined by negative staining or cryo-electron microscopy; data not shown) and did not affect viral genome copy number.

10.1128/mBio.03203-19.4 TABLE S1 IC_50_ and IC_90_ values and cellular toxicity for the set of 17 compounds that were subjected to further analysis. These 17 compounds and the previously published comparison molecule CM9 (also shown) demonstrated a direct and temperature-dependent virucidal effect against clinically relevant strains of virus prior to virus-target cell interaction. Download Table S1, TIF file, 2.6 MB. Copyright © 2020 Marcink et al. 2020 Marcink et al. This content is distributed under the terms of the Creative Commons Attribution 4.0 International license.

**FIG 3:**
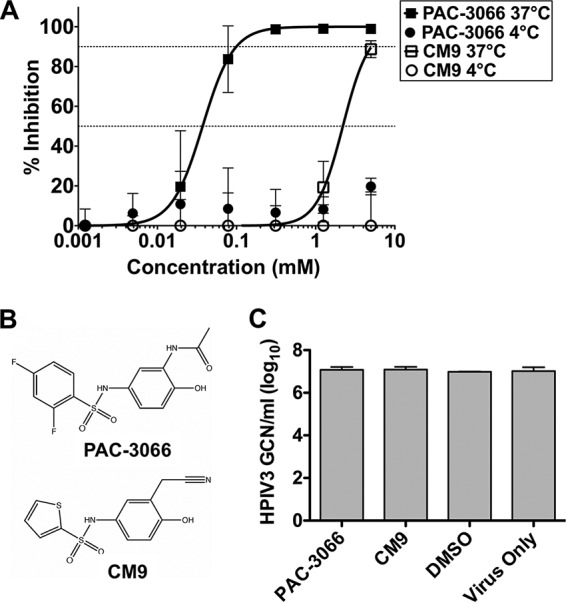
Dose-response of the new preactivating compound compared to that of the previously published prototype. (A) The HPIV3 clinical isolate was incubated with the indicated concentrations of compounds or left untreated for 60 min at 4°C or 37°C. The compounds were removed, and viral infectivity was determined by a plaque reduction assay. The effect of pretreatment with the compounds on infectivity is shown as the level of inhibition of the plaques as a percentage of the number inhibited without preincubation with compounds. (B) Chemical structures of PAC-3066 and CM9. (C) HPIV3 CI-1 eGFP was treated with different concentrations (5 mM, serially diluted 2-fold six times down to 0.078 mM) of the indicated small-molecule compounds for 1 h at 37°C. RNA was extracted from the remaining virus-compound solutions and used for viral genome quantification. The results shown are those obtained after treatment with 5 mM each compound. GCN, genome copy number.

For these experiments, we used recombinant viruses representing a well-characterized clinical isolate of HPIV3 (CI-1) (30) expressing enhanced green fluorescent protein (eGFP) for ease of quantitation. Virions were incubated with the compounds or dimethyl sulfoxide (DMSO) as a control at 37°C for 30 min, and after removal of the compounds, the infectivity of the treated virions was assessed by a plaque reduction assay. Figure 3 shows the results of a dose-response experiment assessing inhibition of viral entry using our previously published compound CM9 (33) and one of the of the compounds with the best activity selected from the 26 compounds, PAC-3066 (Fig. 3A). The IC_50_ of this compound for inhibition of viral entry in this assay, ∼37 μM, was improved 100 times over that of CM9 (33). After treatment with PAC-3066 at even the highest concentrations, the viral genome copy number was unaffected; viruses were treated with concentrations ranging from 5 mM to 78 μM, and no change in genome copy number was detected by quantitative reverse transcription-PCR (Fig. 3C), similar to the results obtained with our proof-of-concept compound, CM9 (33).

The structure of PAC-3066 is similar to that of CM9 (Fig. 3B), and consequentially, most of the docking pose and interactions with HN remain the same (Fig. 2, right). The thiophene ring is replaced with a 3,5-difluoro-phenyl moiety, which is larger and which therefore forms stronger hydrophobic and stacking interactions with H552. The nitrile of CM9 has been replaced by an acetamide, which hydrogen bonds with both K553 and T525. These additions to the binding pose may explain the improvement in the IC_50_ by 100 times.

PAC-3066 and CM9 share a similar chemical moiety that includes sulfonamide between two aromatic rings, where the amine is protonated and can serve as a hydrogen donor. In the docking pose of PAC-3066 and CM9, the amine donates a hydrogen bond to the backbone carbonyl of N551, while the sulfate accepts hydrogen bonds from the backbone amine of K553 and the side chain of S554 (Fig. 2). Sixteen compounds, in addition to PAC-3066 and CM9, inhibited viral infection by HPIV3 (Fig. S1). These do not share the moiety consisting of sulfonamide between two aromatic rings, and except for compound 0434, their IC_50_s were higher (i.e., they have lower activity) than that of CM9. With an IC_50_ of 1.82 μM, 0434 was slightly more potent than CM9 (but much less potent than PAC-3066), possibly due to a unique long polar chain, which is engaged in multiple hydrogen bonds with N551, K553, and D556 in the proposed docking pose.

10.1128/mBio.03203-19.1 FIG S1 Binding poses of additional inhibitors of HPIV3 infection. The ligands are highlighted with purple bonds, while the protein amino acids have orange bonds. The atoms are colored with the CPK coloring convention. Hydrogen bonds between the ligand and the protein are marked as dashed green lines. The images were prepared using the LigPlot+ program. Download FIG S1, PDF file, 2.1 MB. Copyright © 2020 Marcink et al. 2020 Marcink et al. This content is distributed under the terms of the Creative Commons Attribution 4.0 International license.

### Preactivating molecule does not block HN-receptor interaction or neuraminidase activity for lab-adapted or clinical isolates of HPIV3.

**FIG 4:**
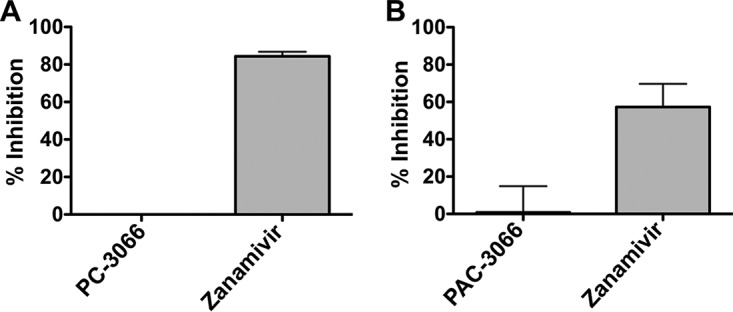
PAC-3066 does not affect HN-receptor interaction or neuraminidase activity. (A) Sensitivity of HN-receptor binding to viral entry inhibitors of cell monolayers expressing clinical isolate HPIV3 HN, incubated with either PAC-3066, zanamivir, or DMSO. Percent inhibition relative to that for DMSO-treated cells is shown. Each point represents the mean from 3 experiments, each of which was performed in triplicate, ± SEM. (B) The inhibition of neuraminidase activity in the presence or absence of the indicated compounds was assayed at 37°C and pH 5 on cell monolayers transiently expressing HPIV3 clinical isolate HN. Each bar represents the results from triplicate experiments, each of which was performed in triplicate, ± SEM.

We assessed the effect of PAC-3066 on HPIV3 HN-receptor binding in a hemadsorption (HAD) assay that quantitates the binding of sialic acid receptor-bearing erythrocytes (RBC) to HN/F-coexpressing cells. For comparison, we used zanamivir, which inhibits HN-receptor binding (35). In contrast to zanamivir, PAC-3066 did not affect receptor binding for clinical strain HNs (Fig. 4A), confirming that the inhibitory mechanism does not involve inhibition of receptor binding. The compound also had no inhibitory effect on viral neuraminidase activity (Fig. 4B), in contrast to zanamivir, which inhibits HPIV3 HN’s neuraminidase (35), indicating that PAC-3066 does not interfere with HN’s primary receptor binding/cleaving site (site I) for HPIV3.

### Synergy of preactivating compound PAC-3066 with receptor inhibitor.

We hypothesized that the combination of zanamivir, which blocks HN’s engagement with cellular receptors by binding to receptor binding site I of HPIV3 HN, and the preactivating compound PAC-3066 could be advantageous. These compounds possess distinct mechanisms of action, and in the context of infection (i.e., exposure to compound in the presence of host cells), receptor blockade could promote the activity of the preactivating compound. We assessed inhibitory activity when the compounds—either each compound alone or the combination—were added after infection, and the inhibitory effect was monitored over time.

**FIG 5:**
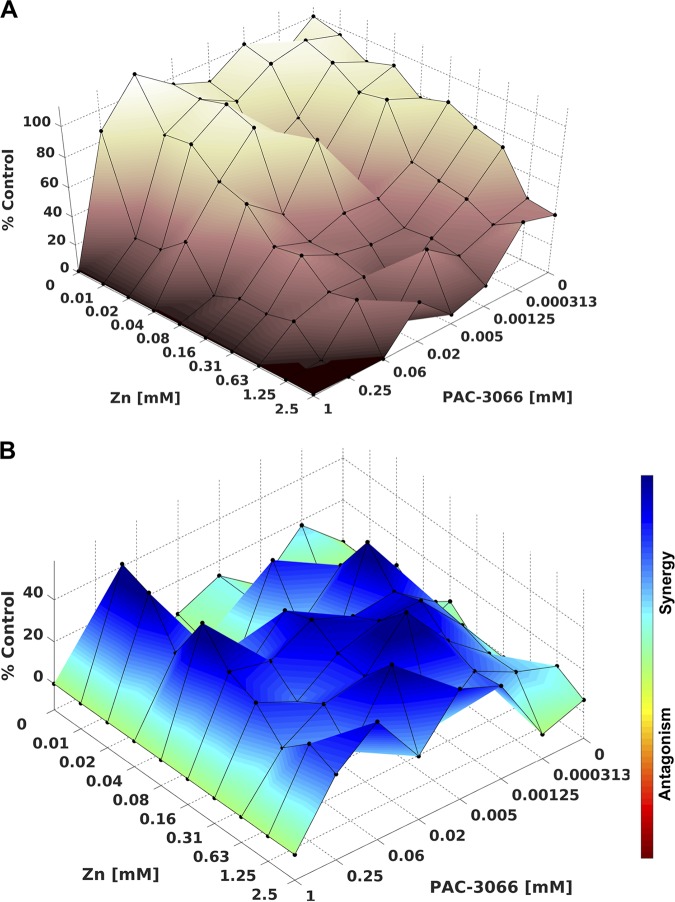
PAC-3066 and zanamivir synergize to inhibit viral infection. (A) Mapping of the dose-response in the presence of zanamivir and PAC-3066. The *z* axis is the percentage of the control response, where the control response is that with no treatment. (B) Synergy plot where the difference between the additive response and the dose-response is the percentage of the control response for the indicated combinations of PAC-3066 and zanamivir.

The effects of the combination of zanamivir and PAC-3066 were assessed by the use of multiple pairwise combinations of zanamivir and PAC-3066 at different concentrations. The dose-response data were processed using the Combenefit program (43) (Fig. 5A). The synergy plot surface shows the additive (green) and synergy (blue) distribution levels (Fig. 5B). The data showed strong synergy between the two drugs over a range of zanamivir/PAC-3066 ratios, but the strongest was at a ratio of 1:1, 1:3, or greater (particularly at a PAC-3066 concentration of 250 μM). At PAC-3066 concentrations below 250 μM, only additive effects were observed (as indicated in the matrix plot showing the quantitation of percent synergy), because the IC_50_ of pure PAC-3066 in these assays was 308 μM. The numbers corresponding to Fig. 5 are shown in Fig. S2; the matrix plot in Fig. S2 takes into consideration the error and shows blue only where there was a significant increase and a low error. Of note, PAC-3066 and zanamivir were added to the virus and cells at 2 h after cellular infection, in contrast to the assay whose results are shown in Fig. 3, where PAC-3066 was added to virus an hour prior to cellular infection. The goal here was to determine whether the combination had the effect of decreasing the replication and spread of virus. In contrast to PAC-3066, which is expected to inactivate virus at a distance from the infected cell, zanamivir does not affect an isolated virus but prevents cell receptor engagement and thereby prevents infection and cell-cell spread. The experimental strategy used here was designed to capture the effect of the combination of molecules during infection, since the compounds were added after infection and inhibition was assessed over a period of time. At higher concentrations of PAC-3066, e.g., 1,000 μM, there was no synergy with zanamivir because there was already a maximal effect of compound and minimal detectable infection, obscuring any additional effect. No significant antagonism was observed.

10.1128/mBio.03203-19.2 FIG S2 PAC-3066 and zanamivir synergize to inhibit viral infection. (A) Matrix plot of the dose-response in the presence of zanamivir and PAC-3066. The color range from dark to light brown corresponds to the percentage of the value for the control, where the lightest brown is the value for the control (no treatment). (B) Synergy matrix plot, where the difference between the additive response and the dose-response is indicated. The color range from red to blue indicates the effect of combinations, where darker blue indicates the highest synergy. Synergy levels over 25 are colored in shades of blue. Significant synergy levels are denoted with indications of significance, determined by a one-sample t test (*, *P* < 0.05; ***, *P* < 0.00001). Download FIG S2, TIF file, 2.8 MB. Copyright © 2020 Marcink et al. 2020 Marcink et al. This content is distributed under the terms of the Creative Commons Attribution 4.0 International license.

### Direct evaluation of mechanism of action of PAC-3066: cryo-electron microscopy detection of the conformational rearrangement in F.

**FIG 6:**
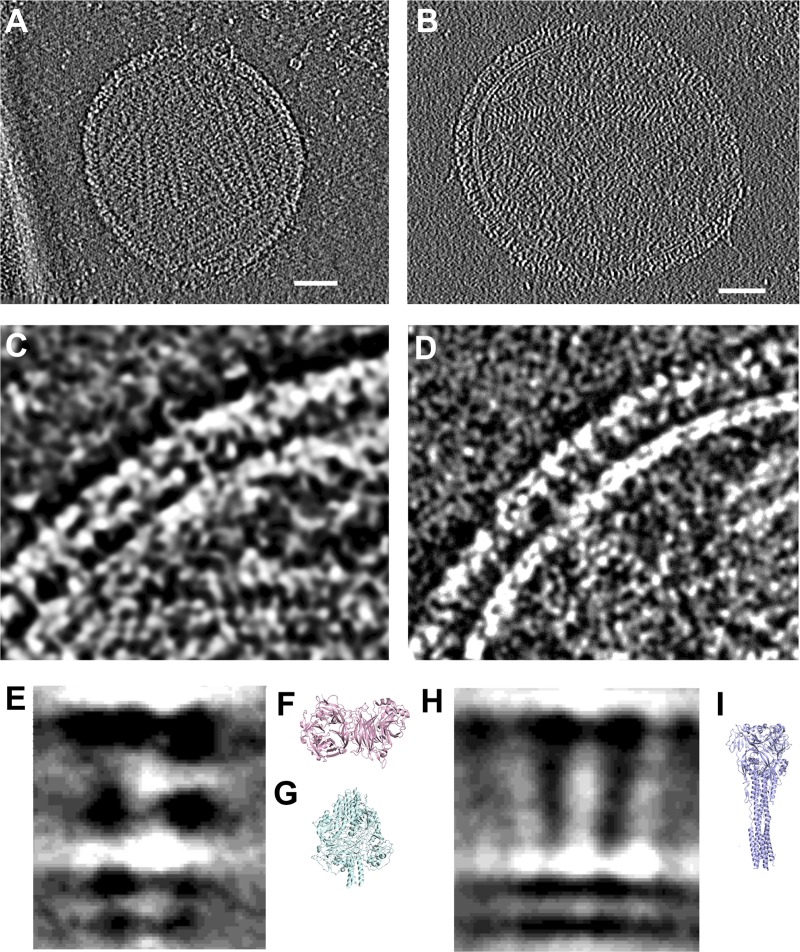
Conformation change in F induced by PAC-3066 at 37°C visualized by cryo-electron tomography. (A, B) Overview of HPIV3 without (A) and with (B) PAC-3066. Bars, 50 nm. (C, D) Close-up of the viral surface glycoproteins without (C) and with (D) PAC-3066. (E) Subtomogram averages from a subset of viral surface glycoproteins where prefusion F can be identified without PAC-3066. (F, G) Structural comparison coordinates of HN (PDB accession number 4MZA) and prefusion F (PDB accession number 6MJZ) are shown in pink and cyan, respectively. (H) Subtomogram averages where postfusion F can be identified after PAC-3066 incubation. (I) Structural comparison coordinates of the postfusion F (PDB accession number 1ZTM) are shown in light blue.

We have previously shown, using indirect biochemical methods (acquisition of protease sensitivity of conformation-specific antibody binding) that the prototype preactivating molecules act by causing the untimely structural rearrangement of the F protein (33, 34). Here, for the first time, we show direct structural evidence that such a molecule promotes the posttriggered state of HPIV3 F in the absence of receptor engagement, using a new cryo-electron tomography method that we developed. Viruses collected directly from the cell culture supernatants of cells after a single round of infection were captured on electron microscopy grids using rat monoclonal anti-HN antibodies; we have found this to eliminate purification steps and yield ultraclean grids with viral particles that are biologically authentic (functional for infection and intact) (44). The viral particles were treated with compounds at 4°C and 37°C prior to being attached to the grids. Using this approach, we obtained tomograms of HPIV3 virions showing the densities for HN and F along with the viral membrane at a level of resolution that allows the specific conformations of F to be distinguished. Here, we determined whether, in the presence of these small molecules, HN and F are in contact and F is converted to its postfusion state. The virions on the grids were incubated without (Fig. 6A) or with (Fig. 6B) 250 μM PAC-3066 at 37°C, and no differences in the overall morphology of the virus were observed. However, the surface glycoprotein densities were altered specifically in the presence of PAC-3066 (Fig. 6C and D). By averaging the results for a subset of surface glycoproteins, we could identify a clear alteration in the conformation of F to the postfusion state on the particles treated with PAC-3066 at 37°C. Without PAC-3066, subaverage densities corresponding to HN and F could be identified (Fig. 6E). The Protein Databank (PDB) structures of HN (Fig. 6F) and prefusion F (Fig. 6G), both of which are globular, precisely matched the subtomogram density shapes. With the addition of PAC-3066, the subaverage prefusion F density was lost and densities consistent with the elongated structure of the postfusion F (Fig. 6I) could be identified. At 4°C, F did not activate under either condition, as expected, since F cannot undergo a conformational change at that temperature (33, 34); there was no difference between the treated and untreated virions at 4°C (Fig. S3).

10.1128/mBio.03203-19.3 FIG S3 Viral particles and their surface glycoproteins in the absence and presence of PAC-3066 at 4°C visualized by cryo-electron tomography. (A, B) Overview image of HPIV3 without (A) and with (B) PAC-3066 at 4°C. Bars, 50 nm. (C, D) Close-up of the viral surface glycoproteins without (C) and with (D) PAC-3066. Download FIG S3, TIF file, 1.7 MB. Copyright © 2020 Marcink et al. 2020 Marcink et al. This content is distributed under the terms of the Creative Commons Attribution 4.0 International license.

## DISCUSSION

To develop an antiviral strategy for enveloped RNA respiratory viruses, we exploited mechanistic knowledge about the exquisitely sensitive and specific nature of the parainfluenza virus fusion process. We have thereby developed compounds that exert profound virucidal effects both *in vitro*, in physiologically relevant *ex vivo* models (33), and *in vivo* for our prototype compound, CM9 (33), in an animal model (data not shown). Compounds that prematurely activate the viral fusion machinery prior to host cell binding, thus rendering the virus particles uninfectious, have potentially significant clinical applications.

Targeting F activation in the way that we propose may have a key benefit: avoiding problematic antiviral resistance. By activating an irreversible viral process and rendering all affected virions inert, we may reduce the selective pressure on the infecting viruses to evolve resistance. In the event of the emergence of variants, escape mutations in HN that render it insensitive to compounds that engage site II would make HN less able to activate F upon receptor engagement as well, thus impairing viral infectivity. Similarly, an escape variant with an F that is less sensitive to activation by HN will also be ineffective at infecting at the cell surface. We hypothesize that such resistant viruses will be more vulnerable to the host immune system, since prefusion F will be available for a period longer than the period of availability in the case of wild-type viruses, increasing the window of time for the induction and the activity of neutralizing antibodies.

For RNA viruses in particular, a high polymerase error rate contributes to the nimble evasion of antivirals (45–47), making receptor analogs potentially ineffective (37, 38). In the case of influenza virus antivirals, for example, rapid viral evolution has necessitated the repeated development of new antiviral approaches and continued surveillance, especially in children and immunocompromised populations (48, 49). A small molecule targeting F has been found to block measles virus infection *in vitro* by increasing the stability of F’s prefusion state and preventing activation (50). However, resistance to this inhibitor arose quickly (50–54), with a mutated F that was destabilized (50, 51). The resistant F proteins were inherently destabilized (55) and became neuropathogenic *in vivo* (56–58), and their stability was increased in the presence of inhibitor (50, 51). Several small molecules that prevent RSV F refolding have been identified (59); however, resistance emerges readily (60). Mechanistic studies suggest an association between the small molecules and the F protein, with the virus remaining infectious upon detachment of the small molecules (60). The small molecules that we explore here work in the opposite way, by inducing HN to destabilize the F protein to the point where it folds to its postfusion state asynchronously with membrane merger. We hypothesize that this mechanism of action will be the least likely to elicit resistance, and in future studies, we will assess this and also test the hypothesis that if resistance to this destabilizing strategy is elicited, the resulting virus will be difficult to activate by HN and, therefore, will be unfit.

An anti-HPIV3 strategy combining vaccination and therapeutic approaches would be ideal for prophylaxis during exposure of vulnerable individuals. The currently available treatment options—ribavirin and corticosteroids—do not work, with the exception of corticosteroids for HPIV1-induced croup; existing treatment options may also worsen disease (61). For influenza virus, inhibiting the viral neuraminidase led to effective drugs (62), but for HPIV3, we have shown that small molecules inhibiting neuraminidase do not prevent viral replication (35).

A recombinant sialidase that we reported to be a potential antiviral approach for parainfluenza and other sialic-binding viruses (63) is being studied in immunocompromised people (64, 65) (ClinicalTrials.gov registration number NCT01644877), and while removing sialic acid receptors from cell surfaces to prevent viral entry could potentially complement our proposed strategies for HPIV, the sialidase approach raises concerns, since sialidase treatment quickly elicits viral escape mutants that are highly resistant, and infection by these variants may be facilitated by the sialidase drug itself. In fact, variant viruses arising during evolution in infected hosts could actually be helped to enter target cells by this compound (32, 66). Fusion-inhibitory peptides could be used in combination with the preactivating compounds (67–71), since it is likely ideal to use a combination of drugs that target different aspects of the viral life cycle (72–74).

## MATERIALS AND METHODS

### Induced-fit docking.

Induced fit was used to accommodate known inhibitors in binding site II. The protocol (40) initially generates 20 conformations using the Glide standard precision (SP) mode (41) with a 0.50-van der Waals (vdW) radius for receptor atoms. In the next phase, different amino acid conformations were sampled with the Prime program (75) in a 5-Å radius around the 20 ligand poses. In the last stage, the molecules were redocked into each of the 20 refined structures using the Glide SP mode with normal vdW radii.

### MM-GBSA.

Binding energy estimates, obtained using MM-GBSA (76) calculations, were based on the energy calculated with the Prime program for the apoprotein, for the ligand, and for the complex structure, taking into account the electrostatic desolvation free energy penalty within the generalized born approximation (77). The MM-GBSA binding energy (Δ*G*_binding_) was defined as follows: Δ*G*_binding_ = *G*_complex_ – *G*_ligand_ – *G*_protein_.

### Virtual screening.

A total of 3.1 million small molecules were downloaded from the lead-like subset of the ZINC database (42). These molecules were prepared using the LigPrep tool (Schrödinger LLC, New York, NY) (78), which generated tautomeric forms and protonation states stable at pH 7.0 ± 2.0, resulting in 5.7 million isomers for virtual screening. The HN dimer structure was downloaded from the Protein Databank (PDB accession number 4MZA) and prepared using a protein preparation wizard (79) and the recommended protocol. For all docking calculations presented in this paper, i.e., the induced fit and the virtual screening which followed, the receptor was defined by a grid box with 10-Å edges placed with residues H552 and Q559 at its center. For computational efficiency, the *in silico* screen was performed in two consecutive steps. Initially, all molecules were docked using the low-accuracy Glide high-throughput virtual screen (HTVS) mode, followed by more intensive docking of the top 20% results using the Glide SP mode. The top 1,000 results from the second step were visually examined using the Maestro graphical user interface (Schrödinger LLC, New York, NY).

### Cells and viruses.

293T (human kidney epithelial) and CV-1 (African green monkey kidney) cells were grown in Dulbecco’s modification of Eagle’s medium (DMEM; Thermo Fisher) supplemented with 10% fetal bovine serum (FBS) and antibiotics. Stocks of laboratory-adapted HPIV3 were made in CV-1 cells from virus that had been plaque purified four times. Virus was collected at 36 to 48 h postinfection and stored at −80°C. Stocks of recombinant clinical strain viruses were launched with *trans*-complementation by laboratory-adapted HN and F and were prepared in human airway epithelium (HAE) cultures (30). HAE cultures were infected by applying 200 μl of EpiAirway growth medium containing 4,000 PFU of HPIV3 to the apical surface for 90 min at 37°C. Following incubation, the medium containing the inoculum was removed, and the cultures were grown at 37°C and fed each day with 1 ml of medium basolaterally. At 3 to 5 days postinfection, viruses were harvested by adding 200 μl of Opti-MEM medium (Thermo Fisher) apically for 30 min at 37°C. The suspension was then collected, and viral titers were determined by plaque assay as described previously (32). The viral sequence was confirmed by whole-genome sequencing, as previously described (30, 80).

### HAE cultures.

The EpiAirway Air-100 tissue model (MatTek Corporation) consists of normal human-derived tracheobronchial epithelial cells that have been cultured to form a pseudostratified, highly differentiated mucociliary epithelium closely resembling that of airway epithelial tissue *in vivo*. Upon receipt from the manufacturer, the HAE cultures were transferred into six-well plates (containing 1.0 ml medium per well), with the apical surface remaining exposed to air, and incubated at 37°C in 5% CO_2_ as previously described (32).

### Chemicals.

Zanamivir (Acme Biosciences) was dissolved in Opti-MEM medium to a concentration of 50 mM and stored at −80°C. CSC compound derivatives (CM9) were synthesized by Charnwood Molecular Ltd. Stock solutions of 100 mM were prepared in DMSO. PAC-3066 was synthesized by Acme Biosciences. Stock solutions of 5 mM were prepared in DMSO. Stock solutions were stored at −20°C.

### Transient expression of HPIV3 HN and F constructs.

The HPIV3 HN and F genes from laboratory strains and clinical isolate viruses were cloned into pCAGGS as previously described (29). The constructs used in the assay for F protein triggering, HN-N-Venus and F-C-cyan fluorescent protein (CFP), were prepared as previously described (15). Transfections were performed with the Lipofectamine 2000 reagent (Invitrogen), according to the manufacturer’s instructions.

### Plaque reduction assay.

The effects of the compounds on plaque number were assessed as described previously (34, 35, 81). Briefly, CV-1 cell monolayers were infected with 100 PFU of the indicated virus in the presence of serial dilutions of the indicated compounds. After 90 min, the plates were overlaid with Avicel microcrystalline cellulose (gift from FMC Biopolymer); 24 h later the agarose was removed, the cells were fixed, and the fluorescent plaques (or single infected cells for the clinical strain) were counted.

### Hemadsorption assays.

Hemadsorption (HAD) was performed and quantified as previously described (13). Briefly, the growth medium was aspirated from HN-transfected 293T cell monolayers in 48-well BioCoat plates (Becton Dickson Labware), the medium was replaced with 150 μl of CO_2_-independent medium (pH 7.3; Gibco) containing different concentrations of compounds and 1% RBC in serum-free CO_2_-independent medium, and the plates were placed at 4°C for 30 min. The wells were then washed three times with 150 μl cold CO_2_-independent medium. The bound RBCs were lysed with 200 μl RBC lysis solution (0.145 M NH_4_Cl, 17 mM Tris HCl), and the absorbance at 405 nm was read using a Tecan M-1000 Pro microplate reader.

### Measurement of neuraminidase activity.

Assays for the measurement of neuraminidase activity were performed in transiently transfected 293T cell monolayers, as described previously (13, 34, 82). Briefly, 293T cells expressing HN were added to 96-well plates in CO_2_-independent medium at pH 5.0. After adding reaction mixtures containing 20 mM 2′-(4-methylumbelliferyl)-alpha-d-*N*-acetylneuraminic acid (Toronto Research Chemicals Inc.) substrate and different concentrations of the compounds, the plates were incubated at 37°C for 1 h. Throughout this period, the fluorescence resulting from hydrolysis of the substrate was read at a 365-nm excitation wavelength and a 450-nm emission wavelength using a Tecan M-1000 Pro microplate reader.

### Virucidal assay.

Aliquots of HPIV3 CI-1 eGFP or HPIV3 CI-1 HN H552Q eGFP preparations (10,000 PFU per sample) were incubated for 30 min at 37°C in Opti-MEM medium supplemented with the indicated compounds or DMSO. After incubation, the samples were diluted with Opti-MEM medium and cleared of compounds using Ultrafree MC filters (Millipore). The viral particles were then collected from the filters in Opti-MEM medium, and their infectivity was determined by a plaque reduction assay. Using a one-step quantitative real time-PCR HPIV3 detection kit (Primer Design Ltd.) and Realplex2 Mastercycler (Eppendorf), the total amount of viral RNA in each sample was determined (34). For negative controls, untreated viruses (CI-1 wild type or CI-HN-H552Q) were incubated with an equivalent volume of DMSO under the same conditions. Following incubation, the resulting solutions were used to inoculate Vero cells to assess viral infectivity.

A 1:20 dilution of the molecule-treated solutions (∼500 PFU virus/maximum of 0.625 mM small molecule) was used to infect Vero cells and serially diluted 3 times 1:5 (required for discerning the viral titer via limiting dilution). The virus was left to incubate at 37°C for 90 min, prior to removal and replacement of the medium (DMEM with 1% penicillin-streptomycin and 10% FBS).

Infection was imaged at 24 h postinfection by bright-field and fluorescence microscopy, and cells were fixed at 48 h postinfection (1:2,000 nuclear staining with DAPI [4′,6-diamidino-2-phenylindole] and imaging using an InCell analyzer).

### Cell toxicity assay.

Vero cell monolayer cultures were exposed to the compounds at a 1 mM concentration and incubated for 1 h at 37°C, followed by performance of a standard 3-(4,5-dimethyl-2-thiazolyl)-2,5-diphenyl-2Htetrazolium bromide (MTT) viability assay.

### Quantitative PCR for assessment of viral genome copy number following compound treatment.

HPIV3 CI-1 eGFP was treated with different concentrations (5 mM, serially diluted 1:2 six times down to 78 μM) of compounds for 1 h at 37°C in Opti-MEM medium. In tandem with the assessment of viral infectivity, RNA was extracted from the remaining virus-compound solutions and used for viral genome quantification. Quantitative PCR, using SYBR green fluorescent dye as a marker for amplification, was performed.

### Synergy analysis of PAC-3066 with a receptor inhibitor.

Vero cells were infected with mCherry-HPIV3 for 2 h, after which the cells were overlaid with various concentrations of PAC-3066 and zanamivir and incubated at 37°C for 3 days. Inhibition of viral spread was determined by analyzing the reduction of fluorescence. Dose-response curves and synergy analysis were performed with the Combenefit software package, and the results were visualized with MATLAB software (43).

### Cryo-electron microscopy grid preparation.

HPIV3 strains in cell culture supernatant were incubated for 30 min at 37°C in Opti-MEM medium with and without PAC-3066. Prior to sample incubation with grids, lacey carbon grids containing a thin carbon layer (Ted Pella) were plasma cleaned. The grids were subsequently coated with anti-HN antibodies for 10 min at 25°C and blotted (44). The grids were placed on top of the samples and incubated at 4°C for 30 min. The grids were washed in Dulbecco modified phosphate-buffered saline and vitrified using a Mark IV Vitrobot (FEI Company) at 100% humidity. The grids were stored in liquid nitrogen until they were imaged.

### Cryo-electron tomography collection.

Samples were imaged in a 300-kV Titan Halo electron microscope (FEI Company) equipped with a K3 Summit camera (Gatan) at a pixel size of 1.745 Å. Tilt series were collected at ±16° in 2° increments at a defocus of 3 to 4 μm and a magnification of ×18,000. The total dose was approximately 100 e^−^/Å^2^.

### Tomography imaging analysis.

Tomograms were reconstructed using fiducial-free back-projection in the Etomo software package (83) and analyzed using ImageJ software. At least 10 tilt series were collected for each sample. Viral surface glycoproteins were picked, and the subvolume was averaged and classified using the Dynamo software package (84, 85). Briefly, subvolumes were automatically picked using a surface mesh representation and cropped with a 480-Å box. Particles were subjected to successive rounds of alignment and classification until the particles converged into similar classes of 200 particles each. A representative average was selected for further analysis and presentation.
