# Supplementary figures and images for: Hijacking the Fusion Complex of Human Parainfluenza Virus as an Antiviral Strategy
Source: mBio. 2020 Feb 11;11(1):e03203-19. doi: 10.1128/mBio.03203-19 (PMC7018645; doi:10.1128/mBio.03203-19)

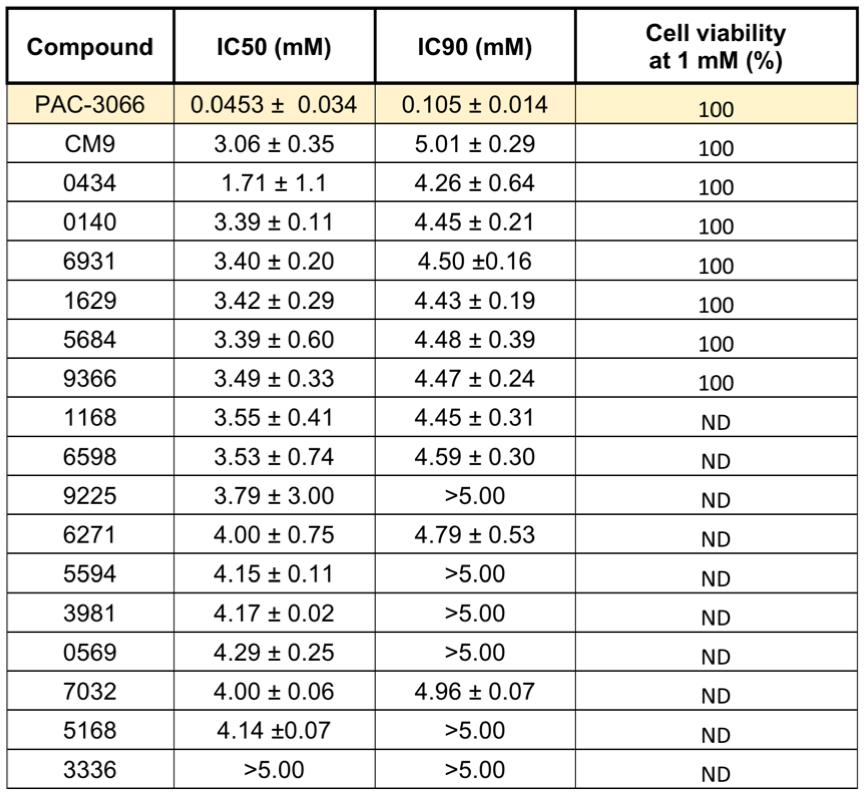

Supplement: TABLE S1 [file mBio.03203-19-st001.tif]

# Fig S1

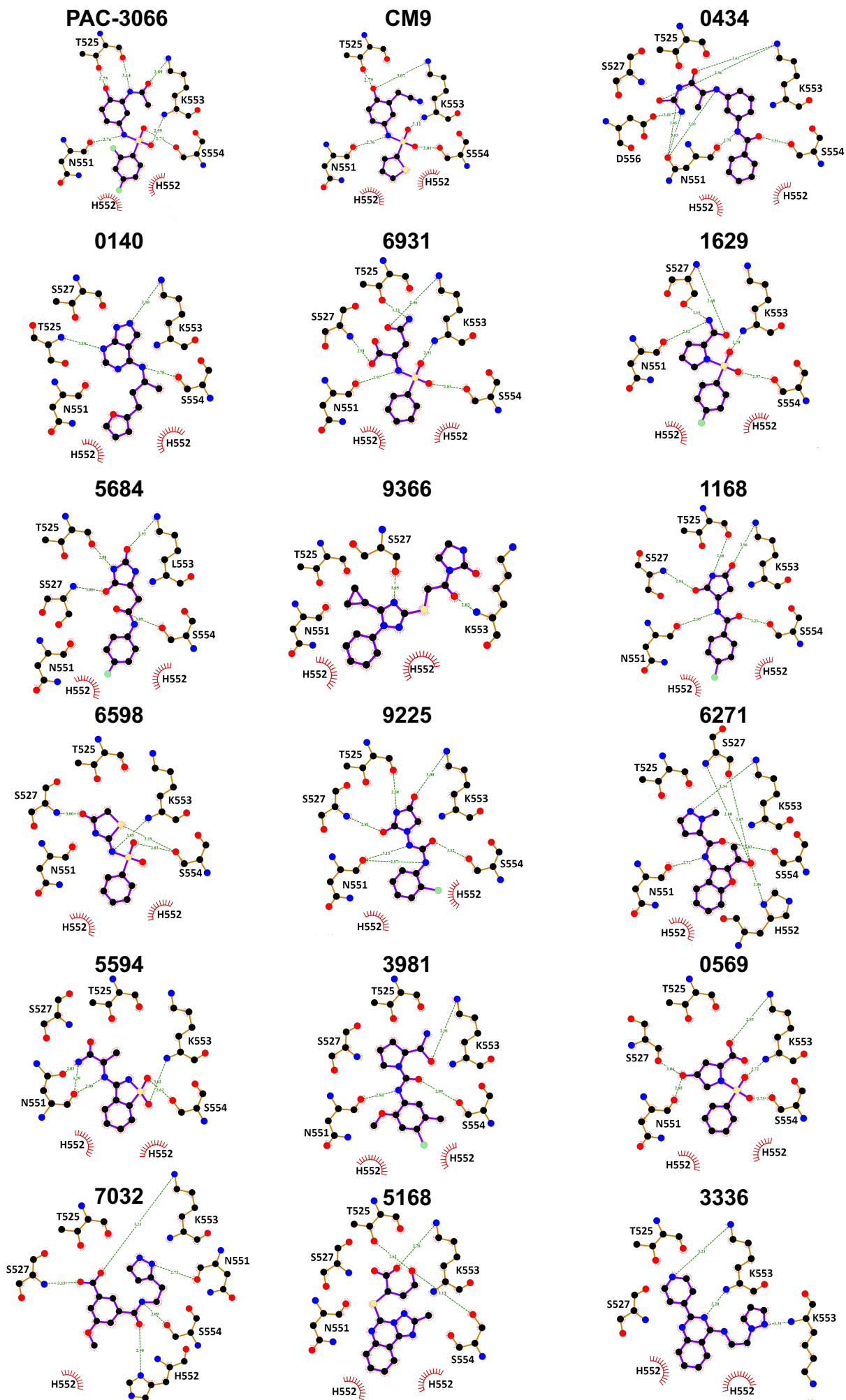

Supplement: FIG S1 [file mBio.03203-19-sf001.pdf]

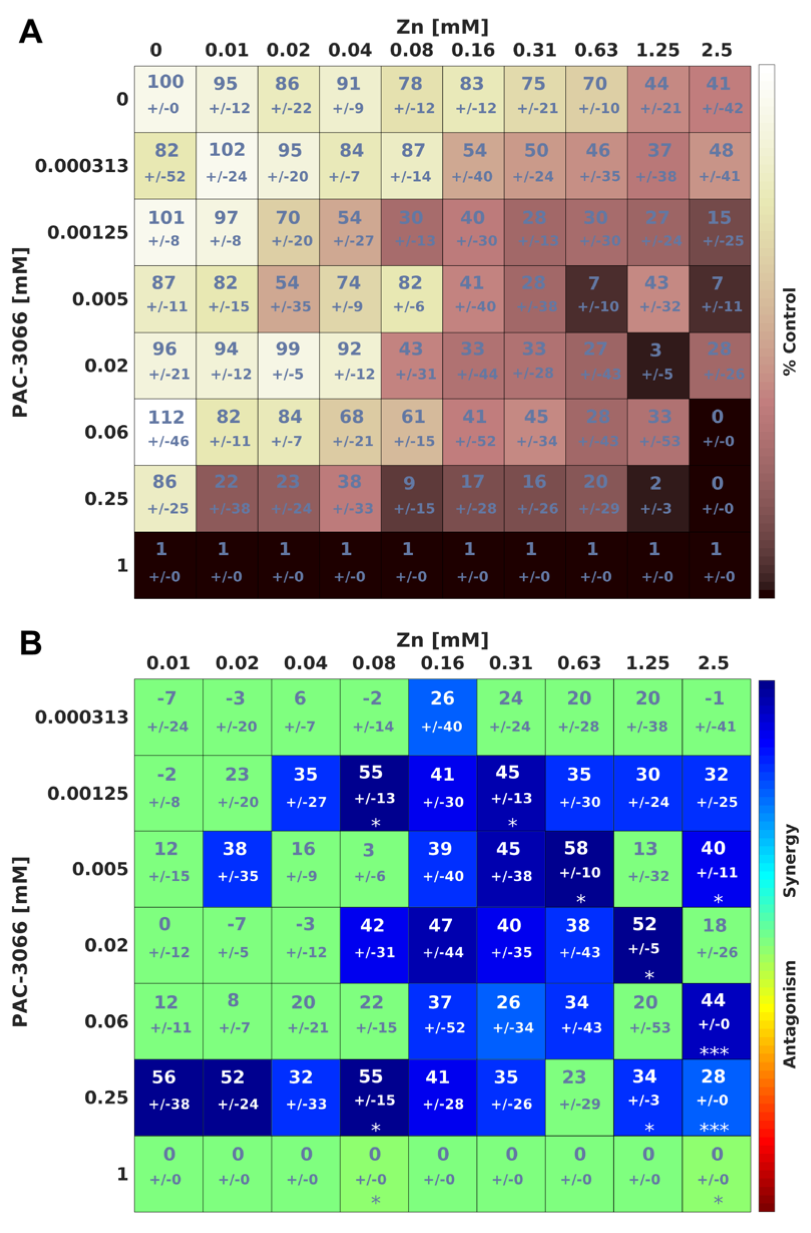

Supplement: FIG S2 [file mBio.03203-19-sf002.tif]

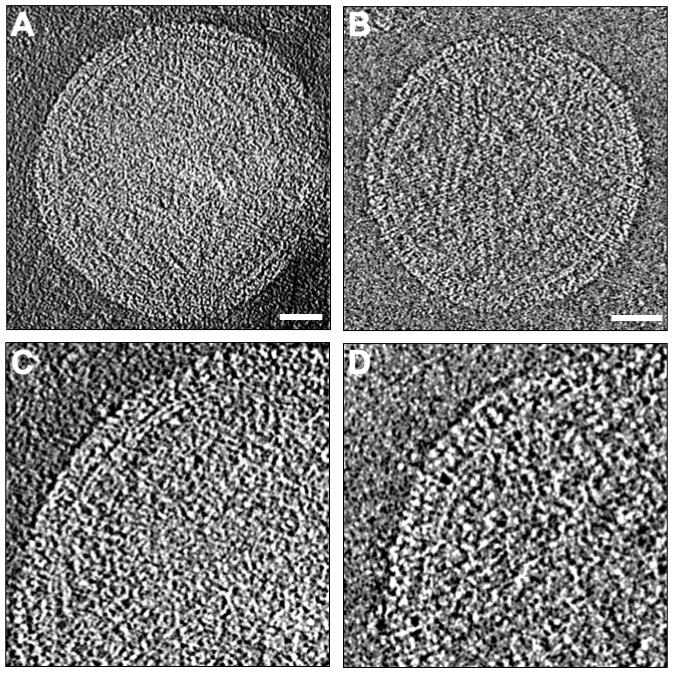

Supplement: FIG S3 [file mBio.03203-19-sf003.tif]
